# Synthesis, Characterization, Antibacterial and Wound Healing Efficacy of Silver Nanoparticles From *Azadirachta indica*

**DOI:** 10.3389/fmicb.2021.611560

**Published:** 2021-02-19

**Authors:** Gandhimathi Chinnasamy, Smitha Chandrasekharan, Tong Wey Koh, Somika Bhatnagar

**Affiliations:** ^1^Plant Transformation and Tissue Culture, Temasek Life Sciences Laboratory, Singapore, Singapore; ^2^Diabetes and Neurodegeneration, Temasek Life Sciences Laboratory, Singapore, Singapore

**Keywords:** antibacterial, antioxidant, wound healing, hydrogel, silver nanoparticles, green synthesis, *Azadirachta indica*

## Abstract

Bacteria are the causative agents of numerous diseases. Ever increasing number of bacterial infections has generated the need to find new antibiotic materials and new ways to combat bacterial infections. Our study investigated *Azadirachta indica* (AI) as an alternate source of antibiotic compounds. Phytochemical and GC-MS analysis revealed presence of flavonoids, phenolic compounds, terpenoids and terpenes. Aqueous extracts of leaves were used to synthesize silver nanoparticles (AI-AgNPs), as established by colorimetric confirmation with maximum absorbance peak at 400 nm. Optimized reaction parameters produced high yield of stable AI-AgNPs, which were characterized by UV-Vis spectroscopy, energy-dispersive X-ray spectroscopy, scanning electron microscopy, and transmission electron microscopy. Results confirmed particle diameter of 33 nm and spherical shape of AI-AgNPs. Fourier transform infrared spectroscopy inferred the presence of functional groups in bioactive constituents involved in conversion of silver ions into elemental silver by acting as capping and reducing agents during formation of AI-AgNPs. X-ray diffraction revealed their crystalline nature. Toxicity studies on *Drosophila* validated normal egg laying capacity and eclosion of F1 generation on AI-AgNPs (100 μg/mL). DPPH (65.17%) and ABTS (66.20%) assays affirmed strong radical scavenging effect of AI-AgNPs (500 μg/mL). The antibacterial activity of AI-AgNPs (1,000 μg/mL) was confirmed by disc diffusion assay with zone of inhibition against *Bacillus cereus* (17.7 mm), *Escherichia coli* (18.7 mm), *Pseudomonas aeruginosa* (10.3 mm), and *Staphylococcus aureus* (17.7 mm). Minimum inhibitory concentration and minimum bactericidal concentration values for AI-AgNPs ranged between 390 and 780 μg/mL. Higher bacterial suppression by AI-AgNPs in comparison with AI-extract was further divulged by prominent damage to the bacterial cell walls, disintegration of cell membranes and outflow of intercellular content as evident in SEM images. AI-AgNPs were loaded on PF127 (biocompatible-biodegradable polymer) to form a viscous, spreadable, hydrogel that demonstrated enhanced antibacterial properties in disc diffusion assay (13–18.7 mm). When topically applied on mice, AI-AgNPs-PF127 hydrogel did not show symptoms of skin irritation. Application of AI-AgNPs-PF127 hydrogel on wound sites in mice, significantly increased the wound contraction rate. Our studies present a simple green route to synthesize AI-AgNPs with enhanced antibacterial and free-radical scavenging efficacy; and AI-AgNPs-PF127 hydrogel as a low-toxic, eco-friendly delivery vehicle with potential in wound healing.

## Introduction

Bacteria are the causative agents of numerous diseases often leading to death and disruption due to damaged crops, spoiled food products, and contaminated equipment (Baranwal et al., 2018). Bacterial infections increase the medical costs and create pressure on health care systems due to long stays in hospitals, treatment failures, persistence of infections and delayed healing of wounds often leading to amputation and increased mortality (WHO, 2020). There is a pressing need to develop new antibiotic material and new strategies to combat bacterial infections. Chemically synthesized drugs have many side effects; hence medicinal plants are being evaluated for curative role owing to their easy availability and non-toxicity for therapeutic use while being effective in curbing bacterial infections (Chinnasamy et al., 2019). Effectiveness of metal nanoparticles (1–100 nm) in combating infectious diseases is an exciting area of research with wide applications (Zazo et al., 2016; Lee et al., 2019). Physical and chemical routes to synthesize nanoparticles involve expensive apparatus and reagents, high voltage, high temperatures, and toxic solvents which leave hazardous residues and by-products raising safety and health concerns for environment and humans (Kumar and Yadav, 2009). Phyto-nanotechnology has gained considerable attention as an alternative, simple, rapid, easily scalable, and cost-effective route where nanoparticles are synthesized using extracts from plants, viruses, algae, fungi, and bacteria (Thakkar et al., 2010; Ahmad et al., 2019). Use of plant extracts has advantage of biocompatibility as they are rich in bioactive compounds which are amicable to extraction by water as an inert solvent and further act as reducing and capping agents in the synthesis of nanoparticles (Noruzi, 2015). Among the different metals in use such as gold, copper, iron, titanium, zinc; silver is the most prevalent one in therapeutic applications owning to inherent antimicrobial properties. Silver nanoparticles synthesized from plants that are ubiquitous in secondary metabolites, have been documented for their intrinsic property of antibacterial inhibition in foodborne pathogens and antibiotic resistant bacteria (Jain and Mehata, 2017; Loo et al., 2018). Among the various drug delivery systems, thermo-sensitive hydrogels are materials of choice for tissue engineering and wound healing applications due to their water holding capacity, uniform dispersion of therapeutic agents and their release in a controlled manner (Huang et al., 2019). Thermo-reversible Pluronic F-127 with sol-gel transition at body temperatures has been found to enhance topical wound healing (Arafa et al., 2018). *Azadirachta indica* (AI) commonly known as neem, is a tropical tree that originated in Indian sub-continent and its distribution has spread worldwide. United Nations acknowledged the importance of this tree and entitled it as “Tree of the 21^*st*^ century.” It is a revered home remedy and find multiple uses as leaf juice to kill intestinal worms, twigs for cleaning teeth, bark paste, and gum for topical application to treat leprosy and skin ailments, seed oil as mosquito repellent and leaves are known for anti-inflammatory, antipyretic, antimalarial, anticancer and antidiabetic properties (Kumar and Navaratnam, 2013; Sarah et al., 2019). In the current studies, we utilized leaves of neem plant for synthesizing silver nanoparticles (AI-AgNPs) and evaluated them for antioxidant activity and antibacterial potency against four species of bacteria. *Bacillus cereus* causes gastrointestinal illness; *Escherichia coli* causes cholecystitis, urinary tract infection, traveler’s diarrhea, neonatal meningitis, pneumonia; *Pseudomonas aeruginosa* survives on medical devices such as ventilators, catheters, and often causes infections in hospital-patients and *Staphylococcus aureus* causes skin infections, bone and join infections, bacteremia, and sepsis. As many basic biological and physiological properties are upto 65% conserved between humans and *Drosophila melanogaster*, we evaluated the toxic response of orally administered AI-AgNPs in *Drosophila.* Thermo-sensitive hydrophilic PF127 was used as a non-toxic biocompatible hydrogel carrier for AI-AgNPs in topical applications on mice skin towards wound healing.

## Materials and Methods

### Materials

*Bacillus cereus* ATCC 14579, *E. coli* ATCC 25922, *P. aeruginosa* ATCC 15442, and *S. aureus* ATCC 23235 were purchased from American Type Culture Collection (Rockville, MD, United States). Leaves of neem were collected from the premises of Temasek Life Sciences Laboratory, Singapore. Chemicals were procured from Sigma-Aldrich, Singapore.

### Identification of Bioactive Compounds (GC-MS Analysis)

To prepare samples for gas chromatography-mass spectroscopy (GC-MS) analysis, neem leaves were lyophilized in liquid nitrogen using a mortar and pestle to obtain fine powder. 1 g of this powder was weighed and dissolved in either 1 mL of hexane or 1 mL of ethyl acetate along with 1 μL (10 mg/mL) of camphor (internal standard). After vortex, the slush was incubated on a horizontal shaker at 30 rpm for 2 h. The mixture was centrifuged at 4,200 rpm for 25 min at 15°C and the separated organic layer was dried in anhydrous sodium sulphate to remove traces of water. For phytochemical analysis the organic extract was transferred into 2 mL glass vial and loaded in a GC system (Agilent 7890A) with a Mass Selective Detector (MSD, Agilent Technologies 5975C Inert XL) and HP-5MS UI column (30 m × 0.25 mm – 0.25 μm). Experimental conditions of the system were as follows: injection volume – 2 μL; splitless injection; oven program 50°C (1 min hold) at 8°C min^–1^ to 300°C (5 min hold). Spectral analysis of data was by MSD Chem Station Data Analysis software (Agilent Technologies).

### Synthesis and Characterization of AI-AgNPs

Nanoparticles were synthesized and characterized according to our previous paper, Chinnasamy et al. (2019). Briefly, washing of leaves under running water ensured removal of dust, pests, and spores, if any. Air dried leaves were homogenized into powder in a blender. Aqueous extract was prepared by adding 10 g of leaf powder to 100 mL of distil water (1:10 ratio) and heating in a water bath at 50°C for 30 min. The solution was cooled to room temperature (RT, 25°C), filtered through Whatman filter paper (No. 1), labelled as AI-extract and used for synthesis of nanoparticles. 5 mL of AI-extract was slowly added into 45 mL of silver nitrate (AgNO_3_, 1 mM) in an Erlenmeyer flask. The resultant mixture was adjusted to pH 7 and incubated on a rotary shaker at 200 rpm, in dark, at RT for 24 h. During this period, visual observations were made to detect any change in colour. Aliquots were taken out at regular intervals of 6 h to measure the absorbance by UV-Visual spectrophotometer (2100 pro UV-Vis, GE). Thereafter, the nanoparticle suspension was centrifuged at 4,500 rpm for 20 min to collect AI-AgNPs as a pellet. To ensure removal of unreacted silver ions and any unbound phyto-constituent, this pellet was washed thrice with distil water, air-dried, and stored at RT for further use. The stability of AI-AgNPs in five different reagents namely distil water, PBS buffer, NaCl (0.9%), Dulbecco’s modified eagle medium (DMEM), and complete medium (CM) was determined by measuring absorbance in wavelength range of 100–900 nm. On-shelf stability in distil water as a storage solution was tested over an additional period of 28 days by measuring absorbance in wavelength range of 100–900 nm.

AI-AgNPs were subjected to UV-Vis spectroscopy (UV1601, Shimadzu), scanning electron microscopy (SEM, JEOL JEM-6360 OLV), energy-dispersive X-ray spectroscopy (EDX), and transmission electron microscopy (TEM, JEOL JEM-1230) for characterization of shape, size morphology and elemental composition. Fourier transform infrared spectroscopy (FTIR, Thermo Fischer Scientific) recorded the absorption spectra in the range of 4,000–400 cm^–1^ to identify the presence of functional groups involved in bio-reduction. X-ray diffraction (XRD, D8 X-ray diffractometer, Brucker BioScience Corporation) of samples exposed to Cu-Kα radiation over an angular range of 20°–80° (2θ) determined the crystalline nature of AI-AgNPs.

### Toxicity Studies of AI-AgNPs on *Drosophila melanogaster*

#### Fly Strain and Medium Preparation

White eyed fly strain, *w*^1118^ (Bloomington stock #3605) was used for the toxicity studies that included viability, development, and egg to adult survivorship. Flies were maintained on standard culture medium (fly food composed of bacto agar, corn meal flour, brewer’s yeast, dextrose and nipagin). The treatment medium consisted of standard culture medium infused separately with AI-extract and AI-AgNPs; both in 5 different doses (10, 25, 50, 100, and 250 μg/mL).

#### Rate of Eclosion

Freshly emerged flies were kept for mating for 24 h in vials containing standard culture medium. The eggs laid in 4 h were transferred on treatment medium (50 eggs per treatment) and reared until eclosed (growth from egg to adult stage). Observations were recorded as percentage eclosion.

#### Egg Laying Capacity

Freshly emerged flies were grown on treatment medium for 10 days. This was followed by mating of flies for 24 h on standard culture medium. The egg laying capacity (of 50 female flies per treatment) was determined by counting the numbers of eggs laid in 4 h.

#### Rate of Eclosion of F1 Flies

Freshly emerged flies were grown on treatment medium for 10, 20, and 30 days. This was followed by mating of flies for 24 h on standard culture medium. The eggs were collected and grown on standard culture medium (50 eggs per treatment) until eclosed to determine the percentage of F1 flies eclosion.

#### TEM Image

The third instar larvae were grown on treatment medium AI-AgNPs (100 μg/mL) and the intestinal midguts were dissected under a microscope and treated with PBS. Samples were fixed in glutaraldehyde (2.5%) for 24 h and ossified with osmium tetroxide (1%) for 4 h. This was followed by dehydration with alcohol series (30–100%, 15 min each) and fixation with epoxy resin for 24 h. Resin blocks were sectioned into 20–30 μm thick tissue slices using an ultrathin tissue sectioner. Sections fixed on copper grid were stained with lead citrate before viewing under TEM to mark the presence of AI-AgNPs.

### Free Radical Scavenging Assay

The free radical scavenging activity was determined by DPPH (2,2-diphenyl-1-picryhydrazil) radical and ABTS {2,2′-Azino-bis-(3-ethylbensothiazoline-6-sulfonic-acid)} radical assays using standard spectrophotometry method. In separate experiments, 100 μL of test samples in different concentrations (100–500 μg/mL) were mixed either with 100 μL of DPPH (0.1 mM) or 100 μL of ABTS master mix (10 mL of 7.4 mM ABTS + 10 mL of 2.45 mM ammonium persulfate). After incubation at room temperature for 30 min in dark, the absorbance (A) of resultant solutions were taken at 515 nm for DPPH and 734 nm for ABTS. A blank (solution without samples) was taken as control and butylated hydroxytoluene (BHT) as a reference. The percentage of free radical scavenging was calculated by following equation:


$$\mathrm{Percentage}\,\mathrm{of}\,\mathrm{radical}\,\mathrm{scavanging}=\frac{\left(A\,\mathrm{of}\,\mathrm{Control}-A\,\mathrm{of}\,\mathrm{Sample}\right)}{A\,\mathrm{of}\,\mathrm{Control}}\times 100$$

### Antibacterial Activity

#### Disc Diffusion Method

Antibacterial activity of AI-extract and AI-AgNPs against four bacterial species was examined by Kirby-Bauer disk diffusion susceptibility test (Bauer et al., 1966). Single colonies of bacteria were picked and incubated overnight in Mueller-Hinton broth (MHB) at 250 rpm at 37°C. Absorbency of the bacterial suspension was measured at 600 nm by a spectrophotometer and adjusted to a concentration of 1 × 10^6^ CFU/mL. Mueller-Hinton Agar (MHA) plates seeded with bacterial suspension were inoculated aseptically with 6 mm sterile paper discs infused separately with sterile distil water (control), Rifampicin (reference), silver nitrate, AI-extract or AI-AgNPs at a concentration of 1,000 μg/mL. The plates were incubated for 24 h at 37°C and visually examined for zone of inhibition (ZOI) around the discs which was the measure of bioactivity of the disc content. Another set of bacterial plates were similarly prepared to test the effect of AI-AgNPs-PF127 hydrogel.

#### Determination of MIC and MBC of AI-AgNPs

Minimum inhibitory concentration (MIC) was measured as the lowest concentration of AI-AgNPs sufficient to inhibit the growth of bacteria and was tested by broth micro dilution method as recommended by the guidelines of CLSI (2012). The MIC test was performed in a 96 well microtiter plate as described by Klancnik et al. (2010). Bacterial suspensions grown overnight in MHB were adjusted to a concentration of 1 × 10^6^ CFU/mL. From a stock solution of AI-AgNPs (50 mg/mL) serial two-fold dilutions were prepared in MHB. This diluted series were inoculated with bacterial suspensions along with untreated control samples. The final volume in each well was 100 μL. The plates were sealed to prevent evaporation and incubated for 24 h at 37°C. Bacterial growth was measured at 600 nm in a microplate reader (Tecan-Spark). Minimum bactericidal concentration (MBC) endpoint is defined as the lowest concentration of antibacterial agent that completely kills the bacterial population. For determination of MBC, aliquots from each well of overnight microtiter plate were seeded on MHA plates without AI-AgNPs and incubated for 24 h at 37°C.

#### Bacterial Imaging by SEM

AI-extract and AI-AgNP were separately added to bacterial cultures (1 × 10^6^ CFU/mL) in 6-well-plate and incubated for 6 h at 37°C. Plain MHB was used as control. The samples were centrifugated at 3,000 × *g* for 30 min to obtain bacterial pellets. These pellets were thoroughly washed and fixed in glutaraldehyde (2.5%, 30 min), followed by dehydration with ethanol series (30–100%, 15 min each step) and overnight drying in amyl acetate. Samples were then sputter coated with gold and visualized under SEM.

### Wound Healing Study on Mice

#### AI-AgNPs-PF127 Hydrogel Preparation

Pluronic F-127 hydrogel (PF127, 30% w/v) was prepared in ice cold PBS. Treatment hydrogels were prepared by adding three different concentration of AI-AgNPs (0.3, 1, and 3 mg) to PF127 hydrogel. The mixtures were kept on rotary shaker overnight (in cold room), to get a clear solution and obtained gels were stored in refrigerator at 4°C for future studies.

#### Evaluation of Physiochemical Properties of AI-AgNPs-PF127 Hydrogel

Physical appearance of the PF127 and treatment hydrogels were visually observed for characteristics such as colour, homogeneity, and consistency. pH was measured by a standard pH meter after diluting the hydrogels to 1% with distil water. Viscosity of the hydrogels was measured at 4°C by an ostwald viscometer. To analyze the spreadability, 50 μL of hydrogels were pressed slightly between two glass slides and left undisturbed for 10 min (as per El-Houssieny and Hamouda, 2010). Diameter of the spread samples was then measured.

#### Skin Irritation Test

To evaluate toxicity of treatment hydrogel on mice, the skin irritation test was performed following the method of Mohamad et al. (2014). After shaving the hair, 20 μL of pristine PF127 hydrogel and 20 μL of 3 mg AI-AgNPs-PF127 hydrogel was applied on back of mice. Skin responses were noted at 1, 6, 24, and 48 h.

#### *In vivo* Wound Healing Activity

Animal experiments were carried out with an approved protocol from the Institutional Animal Care and Use Committee (IACUC), Nanyang Technological University, Singapore (ARF-SBS/NIE-A0367NTU). Pathogen-free, healthy, adult male albino mice with the body weight around 25–30 g were maintained under 12 h day/12 h night cycle in standard lab conditions. Animals were fed with typical rodent diet and distil water throughout this experiment. After one-week of acclimatization, the mice were anesthetized using 5% v/v isoflurane/air and maintained at 2.5% v/v isoflurane/air. Dorsal skin was shaved, disinfected with 70% ethyl alcohol and 6 mm (diameter) of full thickness excision wounds were created. Mice were divided into four groups each comprising of six mice. Group I: control, Group II: pristine PF127 hydrogel, Group III: 0.3 mg AI-AgNPs-PF127 hydrogel, and Group IV: 1.0 mg AI-AgNPs-PF127 hydrogel. 20 μL of respective hydrogel samples were smeared onto the wound site on 1^st^ day and the wounds were covered with Tegaderm and opsite flexifix transparent wound dressing material. Wound areas were examined on 3^rd^, 5^th^, 7^th^, and 10^th^ day after the surgical procedure and evaluated for the percentage of wound contraction as per the following equation:


$$\mathrm{Percentage}\,\mathrm{of}\,\mathrm{wound}\,\mathrm{contraction}=\frac{\mathrm{Wound}\,\mathrm{area}\,\mathrm{day}\,1-\mathrm{Wound}\,\mathrm{area}\,\mathrm{day}\,n}{\mathrm{Wound}\,\mathrm{area}\,\mathrm{day}\,1}\times 100$$

### Statistical Analysis

All the experiments were repeated thrice. Data was represented as mean value ± standard deviation. The data was analyzed using Student’s *t*-test. Values *p* <0.001 were considered statistically significant.

## Results

### Identification of Bioactive Compounds (GC-MS Analysis)

AI leaves as source of bioactive compounds to synthesize AgNPs made it a simple sustainable method as they were easily available in sufficient quantities throughout the year. The compounds identified by GC-MS analysis have been tabulated in Table 1.

**TABLE 1:** Phytochemical composition of leaves of *Azadirachta indica* identified by GC-MS analysis.

No.	Name of the Compound	Chemical formula	Relative abundance (%) in extractions by		Activity
			Ethyl acetate	Hexane	
1	Ethyl propionate	C_5_H_10_O_2_	39.69	-	Antimicrobial
2	2-Hexenal	C_6_H_10_O	-	4.70	Antioxidant
3	Ethyl butyrate	C_6_H_12_O_2_	2.51	-	Antioxidant
4	Trimethylbenzene	C_9_H_12_	0.75	-	Antimicrobial
5	Methyleugenol	C_11_H_14_O_2_	0.30	-	Anti-inflammatory
6	Dihydroactinidiolide	C_11_H_16_O_2_	0.38	-	Antibacterial, antioxidant
7	2-Butyl-1-octanol	C_12_H_26_O	-	0.40	Antimicrobial
8	Elixene	C_15_H_24_	-	1.43	Antibacterial
9	α-Copaene	C_15_H_24_	-	2.95	Antimicrobial
10	γ-Gurjunene	C_15_H_24_	-	2.16	Antibacterial
11	Caryophyllene	C_15_H_24_	-	23.22	Antimicrobial, antioxidant
12	γ-Elemene	C_15_H_24_	0.44	40.98	Anti-inflammatory
13	Humulene	C_15_H_24_	-	2.73	Anti-inflammatory, analgesic
14	β-Cubebene	C_15_H_24_	-	0.94	Antimicrobial
15	Alloaromadendrene	C_15_H_24_	-	1.46	Antioxidant, antiaging
16	δ-Cadinene	C_15_H_24_	-	0.80	Antioxidant
17	α-Selinene	C_15_H_24_	-	1.35	Antioxidant, analgesic
18	Caryophyllene oxide	C_15_H_24_O	0.40	-	Anti-inflammatory, analgesic
19	Dodecanoic acid, trimethylsilyl ester	C_15_H_32_O_2_Si	2.02	-	Antibacterial
20	Tetradecanoic acid, trimethylsilyl ester	C_17_H_36_O_2_Si	1.44	-	Antibacterial
21	Oleic acid	C_18_H_34_O_2_	1.10	-	Antioxidant
22	Hexadecanoic acid, trimethylsilyl ester	C_19_H_40_O_2_Si	42.75	-	Anti-inflammatory
23	Phytol	C_20_H_40_O	2.27	4.38	Antioxidant, analgesic
24	Phytol, acetate	C_22_H_42_O_2_	-	2.92	Antioxidant
25	Silane, [(3,7,11,15-tetramethyl-2-hexadecenyl)oxy]trimethyl	C_23_H_48_OSi	5.90	-	Antimicrobial
26	Heptacosane	C_27_H_56_	-	1.94	Antibacterial
27	α-Tocopherol	C_29_H_50_O_2_	-	7.64	Antioxidant, wound healing

### Synthesis and Characterization of AI-AgNPs

**FIGURE 1:**
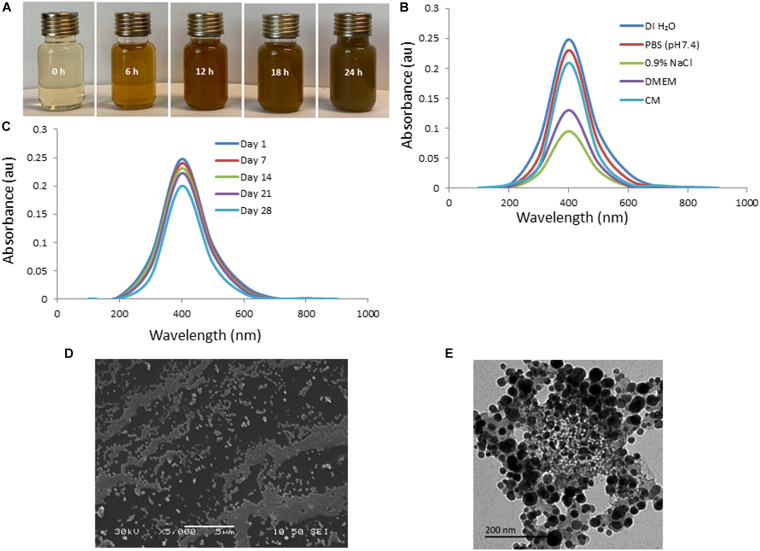
Green synthesis and characterization of AI-AgNPs: **(A)** Change in color during formation, **(B)** Absorption spectra in different test solutions, **(C)** On-shelf stability at various time intervals in UV-Vis spectroscopy analysis, **(D)** SEM image, and **(E)** TEM image.

**FIGURE 2:**
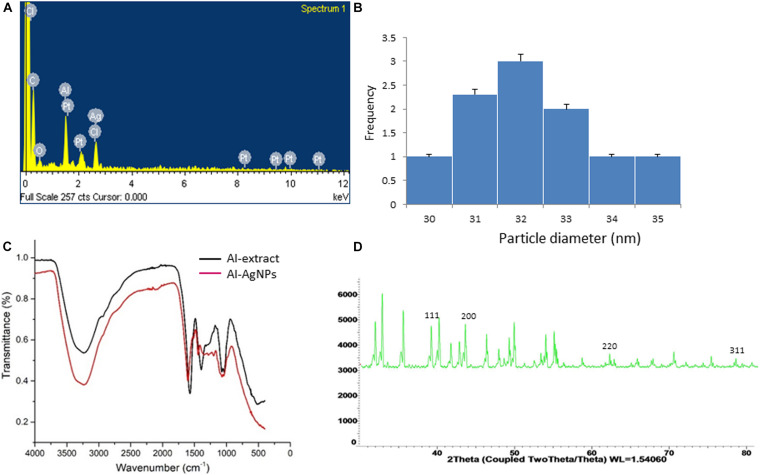
Analysis of surface and content of AI-AgNPs: **(A)** EDX analysis, **(B)** Particle size distribution, **(C)** FTIR spectral analysis, comparison with AI-extract, and **(D)** X-ray powder diffraction analysis.

The efficacy of biosynthesized AgNPs is dependent on the parameters of its preparation like – pH, temperature, concentration of extract, concentration of silver nitrate – which can be manipulated to our advantage to produce AgNPs of well-defined shape, structure, and size distribution to cater to wound healing applications. The change in color of solution from transparent to brown within 5 min of addition of aqueous AI-extract to AgNO_3_ established synthesis of AI-AgNPs (Figure 1A). This was further validated by the presence of a maximum absorbance peak at 400 nm in the UV-Vis spectroscopy. Optimized reaction parameters for synthesis of stable AI-AgNPs were addition of 5 mL of AI-extract to 45 mL of AgNO_3_ (1 mM) at pH 7 and incubation at 25°C in dark at 200 rpm for 18 h. AI-AgNPs were stable in all the five solutions tested (Figure 1B) and during the 28-days test period in distil water at RT (Figure 1C). Results from SEM (Figure 1D) and TEM (Figure 1E) images substantiate AI-AgNPs were of the particle diameter 33.20±3.79 nm and spherical in shape. The presence of elemental silver was represented as strong signal at 3 KeV peak in EDX analysis (Figure 2A) and the particle diameter size range was represented in Figure 2B. Fourier transform infrared (FTIR) inferred the involvement of functional groups present in bioactive constituents in conversion of silver ions into elemental silver by acting as capping and reducing agents. Comparative analysis of FTIR spectra of AI-extract and AI-AgNPs (Figure 2C) depict the shift in peaks from 3254 to 3211 cm^–1^ corresponding to NH or OH stretching vibration of amino or phenolic/hydroxyl, from 2,252 to 2,048 cm^–1^ corresponding to –C=C– stretching vibration of alkenes, from 1,612 to 1,550 cm^–1^ corresponding to –C=O– stretching vibration of amides characteristic of –COOH and from 1,408 to 1,445 cm^–1^ corresponding to -N-H- bending vibration of primary amines. The peak obtained around 1,700 to 1,300 cm^–1^ disclosed formation of AI-AgNPs. This result implied that hydroxyl/phenolic, carbonyl, amide and amino groups were involved in the reduction of silver ion to AI-AgNPs formation. The crystalline nature of AI-AgNPs was evident from XRD pattern where four characteristic peaks were observed at 2θ values of 39.24°, 44.12°, 63.52°, and 77.80° corresponding to crystal facets of (1 1 1), (2 0 0), (2 2 0), and (3 1 1) of face-centered cubic silver (Figure 2D).

### Toxicity Study of AI-AgNPs on *Drosophila*

#### Rate of Eclosion

**FIGURE 3:**
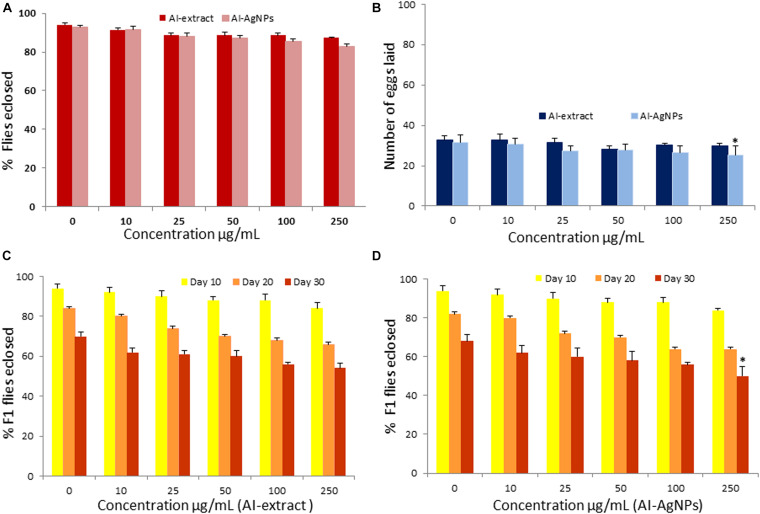
Toxicity effect of AI-AgNPs on *Drosophila*: **(A)** Flies eclosion, **(B)** Egg laying capacity, **(C)** F1 Flies eclosion in AI-extract, and **(D)** F1 Flies eclosion in AI-AgNPs (^∗^*p*≤0.001 considered statistically significant).

To test whether the AI-extract and AI-AgNPs have any adverse effects on physiology and development, we grew *Drosophila* on treatments media containing five different doses of the preparations. The adult flies eclosion rate confirmed that the changes in treatments upto 250 μg/mL were insignificant when compared with the control (Figure 3A).

#### Egg Laying Capacity

No significant change in egg laying capacity of flies was observed in treatments upto 100 μg/mL. *p* ≤ 0.001 significant difference was observed in 250 μg/mL of AI-AgNPs treated group as compared with control groups (Figure 3B). Therefore, the treatment did not affect reproductive functions.

#### Rate of Eclosion of F1 Flies

Percentage eclosion of F1 adult flies did not unfurl any significant difference in the treated and control groups on the 10^th^, 20^th^, and 30^th^ day in medium with up to 100 μg/mL of AI-extract or AI-AgNPs. However, at 250 μg/mL AI-AgNPs treated group shows significant difference (*p* ≤ 0.001) in the F1 flies eclosion rate as compared with the control group on 30^th^ day (Figures 3C,D). Hence, AI-extract did not affect development and overall viability at all tested doses, while AI-AgNPs showed no adverse effect at doses below 250 μg/mL.

#### TEM Image

**FIGURE 4:**
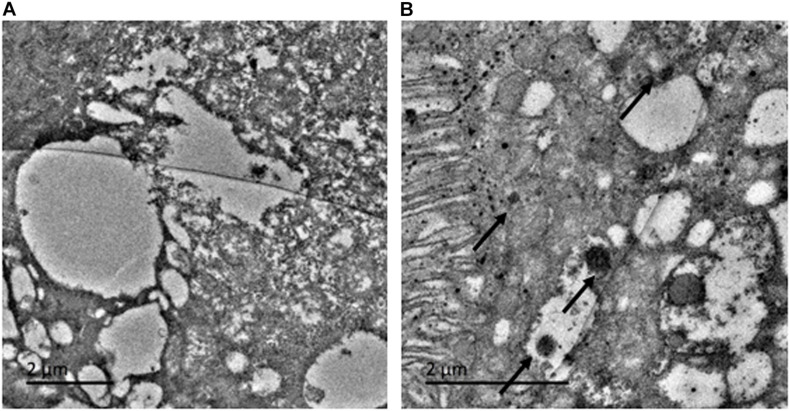
Toxicity effect on *Drosophila* in larval intestinal midgut: **(A)** Control and **(B)** AI-AgNPs treated medium.

To investigate whether the ingested AI-AgNPs were retained by the digestive system of the flies, we examined fly intestines by TEM. The control group was devoid of any particles (Figure 4A). AI-AgNPs were spotted adhering to the microvilli of intestinal lumen and within the cells of intestinal wall in the treated group (Figure 4B), indicating that abundant uptake of the particles had occurred.

### Free Radical Scavenging Assay

Free radical scavenging effect was observed in a concentration-dependent manner. At 100 μg/mL of AI-extract showed 14.45% radical scavenging in DPPH assay and 13.4% radical scavenging in ABTS assay; these values increased to 33.73% and 36.46% at 500 μg/mL. Interestingly, 100 μg/mL of the AI-AgNPs obtained 40.02% radical scavenging in DPPH and 42.71% radical scavenging in ABTS assay, which increased to 65.17% and 66.20% at 500 μg/mL. The results confirmed two-fold increase in radical scavenging for AI-AgNPs as compared to AI-extract (Table 2).

**TABLE 2:** Antioxidant activity of AI-Extract and AI-AgNPs.

Conc (μg/ml)	% DPPH radical scavenging			% ABTS radical scavenging		
	AI-extract	AI-AgNPs	BHT	AI-extract	AI-AgNPs	BHT
100	14.45 ± 1.99	40.02 ± 1.57	60.03 ± 1.65	13.4 ± 1.36	42.71 ± 1.39	61.38 ± 1.12
200	20.46 ± 0.71	47.58 ± 2.65	62.84 ± 1.22	19.82 ± 0.99	49.94 ± 2.31	64.25 ± 2.01
300	24.30 ± 1.21	51.85 ± 1.82	68.19 ± 1.72	26.73 ± 1.13	54.26 ± 2.69	68.62 ± 2.24
400	31.83 ± 1.87	59.43 ± 0.92	78.79 ± 1.16	32.32 ± 2.73	59.92 ± 2.27	77.98 ± 2.11
500	33.73 ± 1.44	65.17 ± 1.21	86.93 ± 1.08	36.46 ± 2.64	66.20 ± 1.52	88.02 ± 1.37

### Antibacterial Activity

#### Disc Diffusion Assay

**FIGURE 5:**
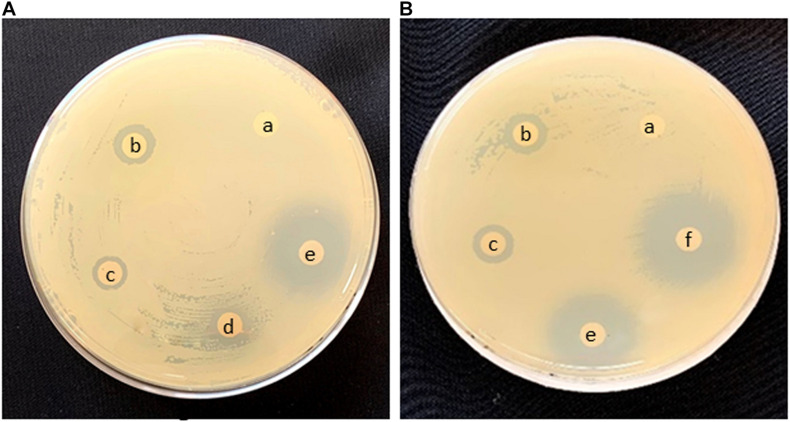
Antimicrobial effect in Disc diffusion assay of **(A)** *S. aureus* and **(B)** *E. coli* **(a)** control, **(b)** rifampicin, **(c)** AgNO_3_, **(d)** AI-extract, **(e)** AI-AgNPs, and **(f)** AI-AgNPs-PF127 hydrogel.

Preliminary screening for bioactivity of AI-AgNPs on agar plates inoculated with a confluent lawn of bacterial cells proved that growth of all the strains was inhibited though to a varied degree. As tabulated in Table 3, diameter of ZOI reports higher inhibition in *B. cereus*, *E. coli*, and *S. aureus* at 17.7, 18.7, and 17.7 mm, respectively as compared to *P. aeruginosa* at 10.3 mm. Figure 5A is representative of the effectiveness of AI-AgNPs in *S. aureus*. Additionally, this antibacterial efficiency was retained and enhanced in the AI-AgNPs-PF127 hydrogel as evident from Figure 5B for *E. coli* and corroborated by diameter of ZOI measured for *B. cereus*, *E. coli, P. aeruginosa*, and *S. aureus* at 18.7, 20, 13, and 20 mm respectively in Table 3.

**TABLE 3:** Determination of diameter of Zone of Inhibition (ZOI), Minimum Inhibitory Concentration (MIC) and Minimum Bactericidal Concentration (MBC) of AI-AgNPs and AI-AgNPs-PF127 hydrogel tested against bacterial species.

Name of the bacterial species	ZOI for AI-AgNPs (mm)	ZOI for AI-AgNPs-PF127 hydrogel (mm)	MIC for AI-AgNPs (μg/mL)	MBC for AI-AgNPs (μg/mL)
*Bacillus cereus*	17.7 ± 1.24	18.7 ± 0.94	390	390
*Escherichia coli*	18.7 ± 1.15	20.0 ± 1.0	780	780
*Pseudomonas aeruginosa*	10.3 ± 0.50	13.0 ± 0.46	780	780
*Staphylococcus aureus*	17.7 ± 0.47	20.0 ± 0.47	390	390

#### MIC and MBC of AI-AgNPs

Disc diffusion assay results were further validated by determining the least inhibitory effect of AI-AgNPs as MIC and the concentration of least biocidal agent required to kill 99.9% of bacteria as MIB when cultured on bacterial media. MIC and MBC values ranged from 390 to 780 μg/mL as represented in Table 3.

#### Mechanism of Antibacterial Action

**FIGURE 6:**
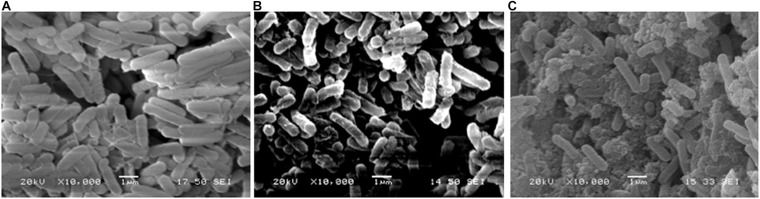
Antibacterial activity in SEM image of *E. coli*: **(A)** control, **(B)** AI-extract, and **(C)** AI-AgNPs.

Bacterial cells observed under SEM, revealed that in comparison with the intact cells in control (Figure 6A) and slightly disrupted cells in AI-extract (Figure 6B); a sub-lethal concentration of AI-AgNPs can result in damaged cell membranes, shrunken cytoplasm and leakage of cell content (Figure 6C).

### Wound Healing Activity

#### Evaluation of AI-AgNPs-PF127 Hydrogel

Due to the presence of AI-AgNPs, the AI-AgNPs-PF127 hydrogel appeared pale ash brown, while the pristine PF127 hydrogel was transparent. pH of the two hydrogels were 5.7–5.8. They exhibited impressive features in terms of sol-gel transition between 22 and 37°C, being liquid at 4°C and hydrogel at 37°C. Viscosity of the hydrogels increased with the increase in temperature. Spreadability of the hydrogels was in the range of 6.0–7.7 cm. Except for slight difference in colour, other characteristics of PF127 were retained in AI-AgNPs-PF127 hydrogel.

#### Skin Irritation Test

AI-AgNPs-PF127 hydrogel did not produce any undesirable side effects such as skin redness, dryness, or flakiness when applied on skin of mice. The skin of both control and treated animals appeared normal.

#### Wound Healing Study

**FIGURE 7:**
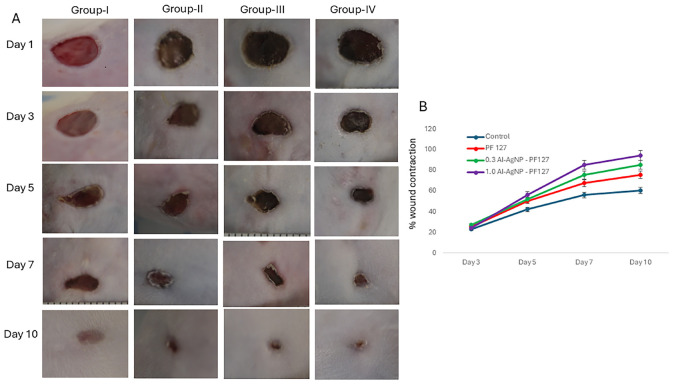
Wound healing process in mice: **(A)** Image represents effect on control (group-I), pristine PF127 hydrogel (group-II), 0.3 mg AI-AgNPs-PF127hydrogel (group-III) and 1.0 mg AI-AgNPs-PF127 hydrogel (group-IV) and **(B)** percentage wound contraction with time.

The quantitative analysis of wound healing involved measuring the initial wound size (1^st^ day) along with healing towards wound closure (10^th^ day). The healing rate in terms of percentage wound contraction was 23.12, 42.33, 56.11, and 60.42 on 3^rd^, 5^th^, 7^th^, and 10^th^ day for control group. Animals treated with pristine PF127 hydrogel showed 25.46, 50.11, 67.54, and 75.77 wound contraction, test group 0.3 mg AI-AgNPs-PF127 hydrogel displayed 27.22, 52.32, 75.44, and 85.52 and test group 1.0 mg AI-AgNPs-PF127 hydrogel reached 24.25, 56.43, 85.23, and 94.54 wound contraction rate on 3^rd^, 5^th^, 7^th^, and 10^th^ day, respectively (Figure 7A). The results showcased near complete wound closure with 1.0 mg AI-AgNPs-PF127 hydrogel on 10^th^ day, thus confirming its healing potential (Figure 7B). 1 mg of AI-AgNPs-PF127 hydrogel treated group had significantly faster healing effect as compared to control group.

## Discussion

Traditional medicinal plants are being explored as source of new drugs against increasing number of antibiotic resistant bacteria (Anand et al., 2019). All parts of a neem plant are used in traditional and folk remedies for a variety of ailments and hence are commercially exploited today in both pharmacology and cosmetic industry. Our preliminary phytochemical screening of neem leaves confirmed the presence of flavonoids, phenolic compounds and terpenoids. GC-MS analysis of AI-extract further revealed elemene, caryophyllene, tocopherol, 2-hexanal, and phytol in extraction with hexane (a non-polar solvent) and ethyl propionate, hexadecanoic acid, trimethylsilyl ester and Silane,[(3,7,11,15-tetramethyl-2-hexadecenyl)oxy]trimethyl in extraction with ethyl acetate (a polar solvent). These phyto-chemicals are known for analgesic, anti-inflammatory, antimicrobial, antioxidant, and wound healing activities. GC-MS analysis of supercritical carbon dioxide extraction of seed and steam-solvent extraction of crude oil from leaves of neem elucidated different phytochemicals (Sonale et al., 2018; Babatunde et al., 2019).

Silver nanoparticles have garnered prominent position in disease management due to their unique properties owing to small dimension and large surface area, mechanical and thermal stability, chemical inertness, electrical conductivity, biosensor, and antimicrobial activity (Rai et al., 2014). The green synthesis of AI-AgNPs was evident from change in colour of solution which is attributed to surface plasma resonance phenomenon. A clear dominant peak at 400 nm in UV-Vis spectroscopy and microscopic evaluation (SEM, TEM) established the particle size (∼33 nm) and spherical shape of synthesized AI-AgNPs, which was in accordance with the literature (Zhang et al., 2016). Optimized conditions of reactants concentration and ratio, temperature, pH, light, and duration resulted in high yield of AI-AgNPs. Quality and quantity of biosynthesized AgNPs depend on reaction parameters and their properties are often governed by the presence of secondary metabolites (Singh et al., 2016). Detection of functional groups such as phenolic/hydroxyl, amide, and amine in FTIR spectra indicated that alkaloids/flavonoids/terpenoids present in AI-extract played the role of reducing agents during the formation of AI-AgNPs. Alkaloids, flavonoids, steroids, terpenoids and tannins are present in neem extract (Thakurta et al., 2007; Sarah et al., 2019); this supported our FTIR findings.

The antibacterial property of AgNPs is harnessed in medical devices, wound dressing, and food packaging; thus, it is important to analyze their toxicity and determine the safe dosage to mitigate any detrimental effect on health. *Drosophila* has been increasing utilized as an *in vivo* model organism for studying human diseases, as nearly 75% of human disease-causing genes have a functional homolog in *Drosophila*; in addition, it has a short life cycle, high reproduction rate, and ease of cultivation in a cost-effective manner (Ong et al., 2015; Vecchio, 2015). Effect of ingesting chemically synthesized AgNPs, studied on emergence of adult flies and lifespan of their progeny in a dose dependent manner, observed normal behavior until 50 μg/mL of AgNPs (Raj et al., 2017). In our toxicity study, exposure to doses upto 100 μg/mL did not have any significant effect on survival, development, and growth of parent as well as F1 generation flies. Similar effect of chicken egg-protein based AgNPs (100 μg/mL) on hatchability, viability, development, and pigmentation was reported by Thiyagarajan et al. (2018). This proved the enhanced safety in using biologically synthesized AgNPs over chemically synthesized AgNPs. Our TEM image displayed AI-AgNPs attached to the intestinal microvilli, lumen, and cytoplasm of midgut epithelial cells of larvae. Though the presence of AI-AgNPs did not result in any obvious phenotypic deviation in features from those of control flies, in future, we plan to further extend our toxicity study on reactive oxygen species (ROS) at gene expression level.

Neem extract is rich in phytochemicals which are inherent hydrogen donors, oxygen quenchers and redox agents that can deactivate free radicals or activate antioxidant enzymes to disrupt this oxidation reaction chain (Septiyani and Wibowo, 2019). The nature of DPPH and ABTS to easily accept a hydrogen molecule or electron from an antioxidant moiety under stable conditions was exploited to determine the radical scavenging activity of AI-extract. When AI-AgNPs were biosynthesized with polyphenol rich AI-extract, their ability to reduce free radical scavenging enhanced up to two-fold in a dose dependent manner.

The synergistic interactions between the Ag^+^ ions and phytochemicals present in the plant extract resulted in formation and stability of bioactive AI-AgNPs molecules that displayed better antibacterial efficacy than AI-extract. AI-AgNPs are easily penetrable into the bacterial cell wall due to their small size and larger exposed surface area for interaction with cell wall components. Morones et al. (2005) suggested that the amount of AgNPs present on and within the bacteria could be explained by the alterations produced by the AgNPs on the membrane morphology of the bacteria. Bacterial cell machinery comprises of sulphur and phosphorus moieties that are basic in nature. The affinity of acidic silver to these molecules present in cellular matrix within bacterial proteins and DNA, binds with oxygen molecules to form sulphydryl groups (S-H) which accelerates the disintegration of respiratory and replication framework of the pathogen resulting in cell death. Proteomic analysis of silver regulated membrane proteins in *P. aeruginosa* exposed to silver nitrate and AgNPs proved that the silver binding proteins for both AgNPs and Ag^+^ ions had a similar pattern, however, the bio-uptake of Ag^+^ ions and the accumulation of ROS was found to be greater in cells exposed to AgNPs (Yan et al., 2018). Though the mechanism of action of both Ag^+^ ions and AgNPs are similar in nature the effectiveness on target site corresponds to the lower concentration of AgNPs required leading to less agglomeration. MIC and MBC values are estimated to determine the inhibitory capability of AgNPs against a test organism. Micro dilution assay of AgNPs provides a better understanding of its bioactivity due to the dispersed AgNPs in a culture broth in comparison with the diffused AgNPs in a disc diffusion agar plate. It also functions as resistance surveillance especially in antibiotic resistant bacteria (Wiegand et al., 2008). In our study, MIC and MBC values of AI-AgNPs depicted a concentration dependent growth inhibitory effect on all the four strains of bacteria. SEM images of bacterial cells depicted morphologically deteriorating cell structures with dismembered bacterial bodies and increased oozing cytoplasmic contents from control to AI-extract to AI-AgNPs treatment. GC-MS results comprehended a polyphenol rich plant extract formulate an intact, stable polyphenol-nanoparticle conjugate that can adsorb oxygen molecules to release silver ion to adhere on bacterial membrane. The underlying mechanism of dispersion of silver ions by AgNP was illustrated to that of a Trojan-horse-type model that continuously releases Ag^+^ ions from the conjugate matrix into the pathogen (Hsiao et al., 2015). Comparative analysis of bio-uptake of Ag^+^ ions in AgNP treated *E. coli* cells found a higher density of internalized Ag^+^ ions and ROS than extracellular. The internalized Ag^+^ ions hamper the respiratory chain and induces oxidative stress in the bacteria by disruption of membrane-specific enzymes, peroxidation of lipids and development of structural lesions in DNA molecules (Long et al., 2017). Various antibacterial mechanism of action of AgNPs can be summarized as cell membrane adhesion and damage, generation of ROS and cell stress, loss of stability of cellular proteins and RNA, leakage of DNA from nucleus, and alternation of cell signaling pathway (Baranwal et al., 2018).

Untreated wound is susceptible to infections caused by bacteria such as *S. aureus.* Broad-spectrum antimicrobial activity of AgNPs has stimulated the development of AgNPs-based dressing for wound healing. Tian et al. (2007) reported that AgNPs were able to treat inflammation through cytokine modulation and induce wound healing with decreased scar formation. Frankova et al. (2016) also substantiated that AgNPs treated group displays decreased release of growth factors and inflammatory cytokines (which are secreted from immune cells), in human dermal keratinocytes. Our studies proved the potential for AI-AgNPs in bacterial cell disruption which together with its free-radical scavenging ability can be utilized for development of wound dressings. ROS is actively involved in wound healing, when present in low concentrations it fights the invading microbe, however, an imbalance in the oxidative-antioxidant respiratory system can result in excessive production of ROS (Sanchez et al., 2018) and an over accumulation in cells can be antagonistic in would healing. Thus, we tested AI-AgNPs in a dose-dependent manner. Conventional dry dressings material such as gauze, plasters, bandages are making way for plant- based wound dressings incorporated films, foams, and gels (Krishnan and Thomas, 2019). Pluronics (Poloxamers) is an exciting thermosensitive polymer that has a critical solution temperature (CST) below the human physiological temperature thus, it exists as a gel state on the body at 37°C. Being a biocompatible and biodegradable polymer material, with excellent mechanical and thermo-sensitive properties, PF127 hydrogel finds applications as drug carriers in cancer and skin diseases (Akash and Rehman, 2015; Chatterjee et al., 2019). Measurement of viscosity is an important parameter in gel preparations meant for topical medication of wound to complete the filling (Mekkawy et al., 2013). AI-AgNPs-PF127 hydrogel exhibited similar viscosity as PF127 hydrogel. This indicated inclusion of AgNPs with PF127 polymer linkage of the formulated hydrogels did not alter flow behavior. Uniform distribution of the gel on the skin is dependent on the spreadability. Spreadability of ideal formulation was found to be 5.7–8.6 cm (El-Kased et al., 2017) which was met by AI-AgNPs-PF127 hydrogel. The pH of the AI-AgNPs-PF127 hydrogel was between 5.5-5.8. Commonly used hydrogels for wound healing applications have pH in range 4.3–6.8 (El-Kased et al., 2017). Further, AI-AgNPs-PF127 hydrogel did not cause any skin irritation in mice, which progresses it to the testing on wounds. Healing of the wound is a complex process that involves synchronous arrangement among various chemical constituents to allow reconstruction of the impaired tissues and to repair the normal skin functions (Diegelmann and Evans, 2004). ZOI results confirmed greater magnitude of bacterial inhibition by AI-AgNPs-PF127 hydrogel than AI-AgNPs. Similar result of a higher antibacterial activity of amino acid loaded PF127 than control was explained based on accumulation of intracellular ROS in bacterial cells (Santos et al., 2020). AI-AgNPs-PF127 hydrogel is able to restrict the infectivity of both Gram positive and Gram negative bacterial types and provide a sterile environment possible to aid active wound healing. The antagonistic effect of AI-AgNPs-PF127 hydrogel on bacterial growth and survival, couple with timely release of AI-AgNPs upon application make it feasible as a wound dressing. Liu et al. (2010) validated that the topical application of AgNPs stimulated wound-healing process included remodeling, re-epithelialization, and wound contraction processes. According to Diniz et al. (2020), a wound takes more than 14 days to heal completely with application of chemically synthesized AgNPs, loaded on gelatin hydrogel. Interestingly our result showed that AI-AgNPs-PF127 hydrogel led to better healing effect in just 10 days. This was due to continuous release of AI-AgNPs from hydrogel, which timely entered the physiological system and interacted with inflammatory cells present in the wound sites. This slow release ensured no damage to the normal cells while prolonging the wound healing effect.

In summary, our studies have shown green synthesized AI-AgNPs as effective antibacterial and antioxidant agents with enhanced and sustained effects when compared to the leaf-extracts alone. These AI-AgNPs did not have any significant toxic effect on development and reproduction of *Drosophila* when used as a feed additive. The development and application of AI-AgNPs-PF127 hydrogel improved wound contraction rate in mice. AI-AgNPs-PF127 hydrogel did not show any signs of skin irritation in mice. Biosynthesized neem silver nanoparticles loaded PF127 hydrogel as a promising alternative candidate for smart, ecofriendly delivery system in cases of bacterial infections and wound healing.

## Data Availability Statement

The raw data supporting the conclusions of this article will be made available by the authors, upon reasonable request.

## Ethics Statement

The animal study was reviewed and approved by Institutional Animal Care and Use Committee (IACUC), Nanyang Technological University, Singapore (ARF-SBS/NIE-A0367NTU).

## Author Contributions

SB conceived and developed the idea of work and provided guidance. GC, SC, and TK designed the experiments. GC and SC performed the experiments and prepared the manuscript. SB and TK edited the manuscript. All authors reviewed and approved the final manuscript.

## Conflict of Interest

The authors declare that the research was conducted in the absence of any commercial or financial relationships that could be construed as a potential conflict of interest.
