# Correction: Synthesis, characterization, antibacterial and wound healing efficacy of silver nanoparticles from *Azadirachta indica*

**DOI:** 10.3389/fmicb.2026.1817152

**Published:** 2026-03-23

**Authors:** Gandhimathi Chinnasamy, Smitha Chandrasekharan, Tong Wey Koh, Somika Bhatnagar

**Affiliations:** 1 Plant Transformation and Tissue Culture, Temasek Life Sciences Laboratory, Singapore, Singapore; 2 Diabetes and Neurodegeneration, Temasek Life Sciences Laboratory, Singapore, Singapore

**Keywords:** antibacterial, antioxidant, wound healing, hydrogel, silver nanoparticles, green synthesis, *Azadirachta indica*

In the published article, there was an error in Figure 7. An inadvertent duplication of images in Figure 7, specifically in Group-III, Day 3 and Group-IV, Day 5.

The corrected Figure 7 and its caption appear below.

**Figure 7:**
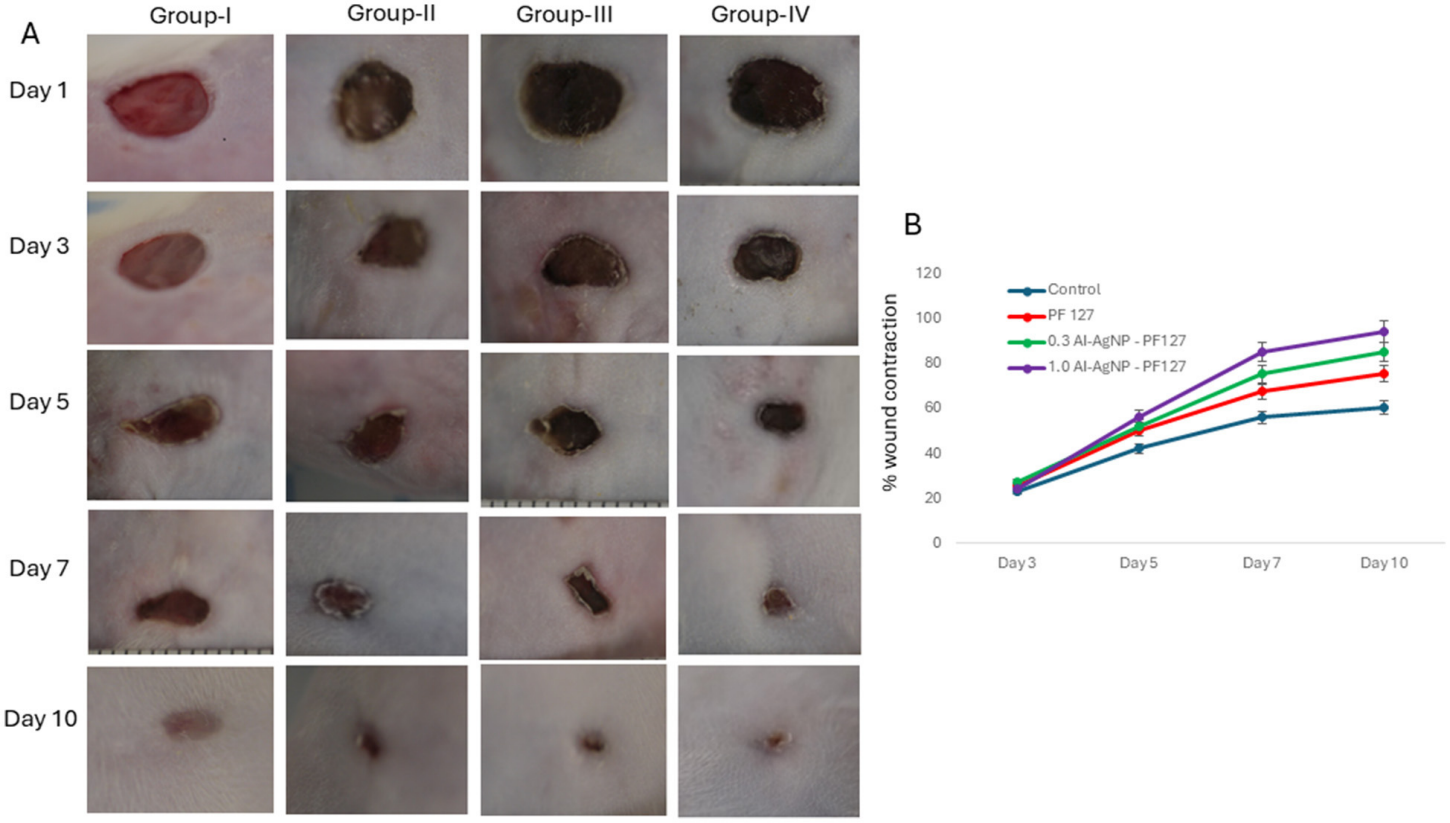
Wound healing process in mice: **(A)** Image represents effect on control (group-I), pristine PF127 hydrogel (group-II), 0.3 mg AI-AgNPs-PF127hydrogel (group-III) and 1.0 mg AI-AgNPs-PF127 hydrogel (group-IV) and **(B)** percentage wound contraction with time.

The original article has been updated.

